# Cytotoxic Polyketides with an Oxygen-Bridged Cyclooctadiene Core Skeleton from the Mangrove Endophytic Fungus *Phomosis* sp. A818

**DOI:** 10.3390/molecules22091547

**Published:** 2017-09-14

**Authors:** Wei Zhang, Baobing Zhao, Liangcheng Du, Yuemao Shen

**Affiliations:** 1 Shandong Provincial Key Laboratory of Synthetic Biology, CAS Key Laboratory of Biofuels, Qingdao Institute of Bioenergy and Bioprocess Technology, Qingdao 266101, China; zhang_wei@qibebt.ac.cn; 2 Department of Chemistry, University of Nebraska-Lincoln, Lincoln, NE 68588-0304, USA; 3 Key Laboratory of Chemical Biology (Ministry of Education), School of Pharmaceutical Sciences, Shandong University, Jinan 250012, China; baobingzh@gmail.com

**Keywords:** polyketide, mycoepoxydiene, cyclooctadiene, endophtic fungus, cytotoxicity

## Abstract

Plant endophytic microorganisms represent a largely untapped resource for new bioactive natural products. Eight polyketide natural products were isolated from a mangrove endophytic fungus *Phomosis* sp. A818. The structural elucidation of these compounds revealed that they share a distinct feature in their chemical structures, an oxygen-bridged cyclooctadiene core skeleton. The study on their structure–activity relationship showed that the α,β-unsaturated δ-lactone moiety, as exemplified in compounds **1** and **2**, was critical to the cytotoxic activity of these compounds. In addition, compound **4** might be a potential agonist of AMPK (5′-adenosine monophosphate-activated protein kinase).

## 1. Introduction

Mangrove endophytic fungi are a rich source of structurally novel and biologically diverse natural products that could be useful in the development of new pharmaceutical agents [1,2]. During the course of our exploration for chemical constituents from the endophytic microorganisms of mangrove, we isolated a series of new compounds with various bioactivities [3,4,5,6,7,8]. Natural products containing an oxygen-bridged cyclooctadiene core skeleton and a *δ*-lactone moiety, as represented by mycoepoxydiene (MED), are recognized as a new class of fungal metabolites [9]. The distinct structural features of this group of compounds have attracted several research groups for total chemical syntheses [10,11]. Previous bioactivity studies suggested that MED could be a promising novel candidate for development of low toxicity antitumor agents [12,13], nonsteroidal anti-inflammatory drugs [14], and anti-atherosclerotic therapeutics [15].

We have been studying a MED-producing strain, *Phomosis* sp. A123, isolated from foliage of *Kandelia candel* (L.) Druce [9,10,11]. Despite the attractive features in structure and activity, MED research suffered from the low yield in strain A123. Subsequently, we used the genome shuffling approach to generate high MED-producing strains, which were screened by the high-throughput screening method, ‘Antimicrobial-TLC-HPLC’ (ATH) [16,17]. These efforts led to several high yield strains for MED analogs, including strain A818, in which the yield increased over 200-fold. The high yield strains produced new analogs in addition to MED. Herein, we describe the isolation, structure elucidation, and bioactivity test of eight natural products of the MED class from strain A818, which was derived from the mangrove endophytic fungus *Phomosis* sp. A123.

## 2. Results

### 2.1. Structure Elucidation

**Figure 1:**
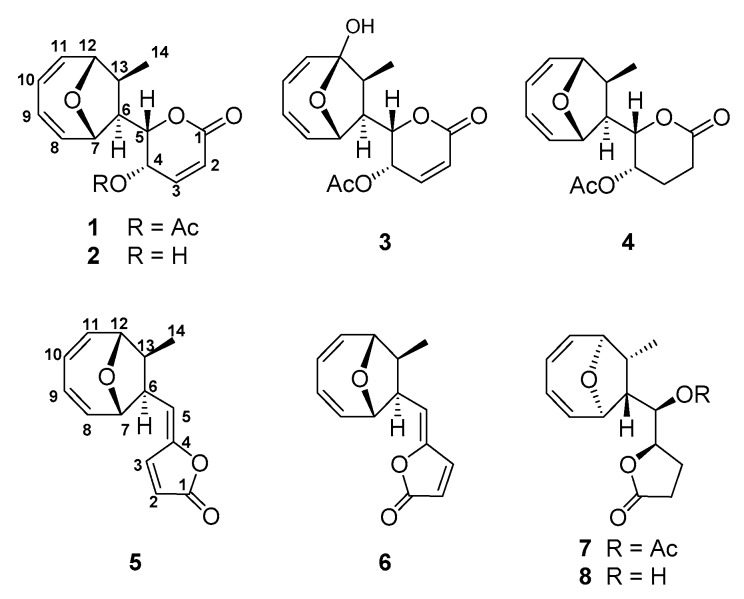
Chemical structures of compounds **1**–**8**.

Compound **1** was obtained as colorless feather-like crystals; its molecular formula of C_16_H_18_O_5_ was determined by ESI-MS ([M + H]^+^ = 291.1225, *calc*. 291.1227), indicating a molecule with eight degrees of unsaturation. The numbers of proton and carbon atoms observed in the ^1^H- and ^13^C-NMR spectra (Table S1) are in agreement with the molecular formula. The DEPT spectrum revealed two methyl groups (*δ*_C_ 20.7, 14.1), two methine groups (*δ*_C_ 52.6, 50.0), four oxygenated methine groups (*δ*_C_ 86.4, 77.6, 75.9, 63.2), six olefinic methine groups (*δ*_C_ 140.2, 137.5, 136.9, 126.3, 125.1, 124.4), and two carbonyl groups (*δ*_C_ 170.0, 162.2) (Table S1). Carbon signals at *δ*125.1 and 140.2 indicated an olefinic bond, which was supported by coupling of protons *δ*6.24 (d, 1H, *J* = 9.7 Hz) and 7.05 (dd, 1H, *J* = 6.0, 9.7 Hz) in ^1^H-^1^H COSY correlation spectrum. By analogy, carbon signals at *δ*_C_ 136.9 and 126.3, *δ*_C_ 124.4 and 137.5 were two adjacent olefinic bonds, which were supported by coupling of protons *δ*6.07 (bdd, 1H, *J* = 5.9, 10.7 Hz) and 5.90 (m), *δ*5.94 (m) and 6.12 (dd, 1H, *J* = 4.5, 7.4 Hz), *δ*5.90 (m) and 5.94 (m) in the ^1^H-^1^H COSY correlation spectrum. These spectroscopic data are consistent with those reported NMR data for MED. Therefore, compound **1** was identified as MED by comparing with reported parameters including configuration [3,9,18] (Figure 1).

Compound **2** was isolated as colorless feather-like or prism crystals, whose molecular formula was determined to be C_14_H_16_O_4_ by HRESI-MS ([M + H]^+^ = 249.1118, *calc*. 249.1121). The IR spectrum showed a hydroxyl, an ester carbonyl, and olefinic bonds at 3395, 1721, and 1592 cm^−1^, respectively. ^1^H- and ^13^C-NMR spectra revealed the presence of a methyl, two methines, four oxygenated methines, six unsaturated methines, and a carbonyl group accounting for 14 carbons and 15 protons (Table S1). These spectroscopic data are consistent with those reported NMR data for deacetylmycoepoxydiene [18,19,20] (Figure 1).

Compound **3** was obtained as a white powder. The molecular formula of C_16_H_18_O_6_ was determined by HRESI-MS ([M + H]^+^ = 307.1175, *calc*. 307.1176). The DEPT spectrum showed the presence of two methyls (*δ*_C_ 19.2, 8.2), two methines (*δ*_C_ 51.3, 49.2), three oxygenated methines (*δ*_C_ 77.8, 71.2, 62.5), six olefinic methines (*δ*_C_ 141.1, 139.2, 137.4, 124.8, 123.9, 123.8), and three carbonyl groups (*δ*_C_ 170.0, 163.2, 106.0) were observed (Table S1). HMBC and ^1^H-^1^H COSY correlation showed that compound **3** shared the same spectroscopic characters with compound **1**, except one quaternary carbon with the chemical shift of *δ*_C_ 106.0. The chemical shift and the observed HMBC correlation between H7 and C12, H10 and C12, H11 and C12, and H14 and C12 supported that C12 in compound **3** was a hydroxylated quaternary carbon. Based on these analyses, the structure of compound **3** was determined as 12-hydroxylmycoepoxydiene (phomoxydiene A) [20] (Figure 1).

Compound **4** was obtained as colorless feather-like crystals with the molecular formula of C_16_H_20_O_5_ as determined by HRESI-MS ([M + H]^+^ = 293.1363, *calc*. 293.1383). ^1^H and ^13^C spectra of **4** shared an overall similarity with those of compound **1**, suggesting that the two molecules were closely related. The only difference between **4** and **1** was the H-2/H-3 double bond in **1** was reduced to methylene groups in **4**, showing that **4** shared the same structure as 2,3-dihydromycoepoxydiene (Figure 1, Table S1) [18].

Compound **5** was obtained as colorless needle-shaped crystals, and the molecular formula was determined as C_14_H_14_O_3_ by ESI-MS data ([M + H]^+^ = 231.1015, *calc*. 231.1016). The DEPT spectrum revealed 14 signals: one carbonyl carbon signal, eight olefinic carbon signals, two methines bearing oxygen signals, two methines, and one methyl group (Table S2). Compared to the NMR data reported for compound 1893A previously isolated from the mangrove fungal 1893 [21], HMBC and ^1^H-^1^H COSY correlations are consistent with those data for 1893A (Figure 1).

**Figure 2:**
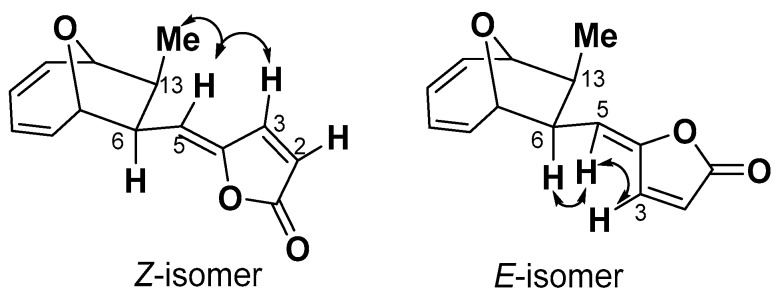
Selected NOESY interactions of compound **5** (*Z*-isomer) and **6** (*E*-isomer).

Compound **6** was obtained as a white amorphous solid, whose molecular formula was determined as C_14_H_14_O_3_ by ESI-MS data ([M + H]^+^ = 231.1013, *calc*. 231.1016). A comparison of ^13^C spectra between **5** and **6** showed that **6** also had one carbonyl carbon signal, eight olefinic carbon signals, two methines bearing oxygen signals, two methines, and one methyl group (Table S2). The clear difference between these two structures was at the chemical shift of C-3, which shifted from *δ*144.3 to *δ*140.1. ^1^H spectrum showed that the coupling constant of *J*_2,3_ = 5.5 Hz of compound **5** changed to *J*_2,3_ = 6.1 Hz of compound **6**. A NOESY signal between H3 and H6 was observed, while no coupling signal was found between H-5 and Me-14 (Figure 2, Table S2). Based on these differences and taking the relative configuration of compound **5** (1893A) into consideration, the structure of **6** was identified as the *E*-isomer of 1893A (phomoxydiene C) [20].

Compound **7** was obtained as colorless needle-shaped crystals, and its molecular formula of C_16_H_20_O_5_ was determined by ESI-MS ([M + H]^+^ = 293.1381, *calc*. 293.1383). ^1^H- and ^13^C-NMR spectra of compound **7** were similar to those of 1893A, and the differences between these two compounds were a series of correlated saturated resonances (*δ*_H_ 2.48, 2.28, 2.06, 4.56, and 5.13), and an acyl methyl signal was observed instead of three olefinic group resonances at *δ*_H_ 7.68, 6.28, and 5.57 ppm in 1893A (Figure 1, Table S2). These spectroscopic data were identical to that of a previously reported compound 1893B [21].

Compound **8** was obtained as a white amorphous solid, and the molecular formula was C_14_H_18_O_4_ as established by HR-ESI-MS ([M + H]^+^ = 251.1275, *calc*. 251.1277). The ^1^H- and ^13^C-NMR spectra of **8** were similar to those of **7**, except the signals for the acetyl group of **7** (Figure 1, Table S2). From the DEPT spectrum, 14 carbon signals were observed, including 1 carbonyl signal, 4 olefinic carbon signals, 4 oxygen-connected methines, 2 methines, 2 methylenes, and 1 methyl group. Both the ^1^H-^1^H COSY correlations and HMBC data supported the structure of **8** to be the deacetylated form of 1893B (phomoxydiene B) [20,21].

### 2.2. Cytotoxicity Study

Compounds **1**−**8** (except compound **3** because only a small amount was obtained) were studied for cytotoxic activity against MDA-MB-435 (a human breast cancer cell line) by 3-(4,5-dimethylthiazol-2-yl)-2,5-diphenyltetrazolium bromide (MTT) assay [22,23]. As shown in Table S3, these polyketide metabolites displayed cytotoxic activity. The results were in agreement with the previous reported data which revealed that compound **1** exhibited more potent activity than compound **2**, while compound **4** was inactive [18]. The result also indicated that α,β-saturated moiety was related to the loss of cytotoxic activity. Compounds **1** and **2** gave the IC_50_ values of 7.85 and 14.61 µM, respectively, against MDA-MB-435, whereas the other compounds showed no significant activity, even at a high concentration (Table S3). Taking the structural features into consideration, the activity results indicate that the δ-lactone moiety may play a more critical role in the cytotoxicity than the γ-lactone in these metabolites.

**Figure 3:**
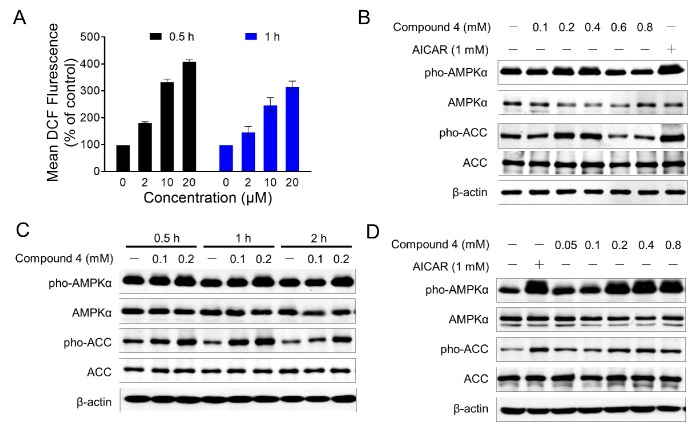
Compound **4** induced the cellular AMPK activation. (**A**) MED induced the generation of intracellular ROS in MDA-MB-435 cells. MDA-MB-435 cells were treated with MED for indicated time, and the intracellular ROS level were determined by flow cytometric analysis with CM-H2DCFDA staining; (**B**) NIH/3T3 cells were treated with indicated concentration of compound **4** for 2 h, and Western blot analyses were performed to determine the indicated proteins level. β-Actin was used as a loading control, and acadesine (AICAR) was used as a positive control; (**C**) Western blot analyses of AMPK and ACC phosphorylation in NIH/3T3 cells treated with compound **4** for indicated time. β-Actin was used as a loading control; (**D**) Western blot analyses of AMPK and ACC phosphorylation in 3T3-L1 cells treated with compound **4** for 2 h. β-Actin was used as a loading control. (Pho-AMPK: phosphorylated AMPK; Pho-ACC: phosphorylated ACC).

Our previous studies showed that MED could induce the arrest of cell cycle and apoptosis [13]. MED could directly activate AMPK in vitro by AMPK kinase assay. However, we did not observe detectable activation of AMPK at the cellular level, by monitoring the phosphorylation of AMPKα and its substrate acetyl-CoA carboxylase (ACC) (data not shown). Indeed, we also found that MED induced the production of the intracellular Reactive Oxygen Species (ROS) in MEDA-MB-435 cells in a dose-dependent manner (Figure 3A). Due to the role of ROS in AMPK activity [24], we speculate that ROS production may impair AMPK activation induced by MED.

Compound **4**, which is inactive in cytotoxicity even at a high dose (up to 800 μM) against NIH/3T3 (mouse embryo fibroblast cell line) (data not shown), offered a new opportunity to study the AMPK activation by new MED analogs. The structure of compound **4** is very close to that of MED. Both contain an oxygen-bridged cyclooctadiene core skeleton and a δ-lactone moiety carrying an acetyl group. The only difference is a double bond in the lactone (α,β-unsaturated δ-lactone in MED). AMPK activation of compound **4** in NIH/3T3 cells was evaluated by monitoring the phosphorylation of AMPK*α* at Thr172 and its substrate acetyl-CoA carboxylase (ACC) at Ser79, with acadesine (AICAR) used as a positive control. Western blot analyses showed that compound **4** dramatically increased the phosphorylation of AMPKα and ACC (Figure 3B). The phosphorylation of AMPK*α* and ACC reached a maximal level at a concentration of 400 μM, whereas no detectable activation was observed in the cells treated with higher concentrations (600 and 800 μM). Furthermore, the activation of AMPK by compound **4** was observed as early as 30 min at 100 μM, and reached a peak value around 60 min without affecting the total content of AMPK (Figure 3C). In addition, compound **4** induced a significant increase in AMPK activation in 3T3-L1 adipocytes (Figure 3D). These results indicated that compound **4** might be a potential AMPK agonist.

## 3. Discussion

The constant emergence of drug resistant diseases and pathogens demands the continuous search for new therapeutic agents. Ideally, the new agents should possess novel features in both chemistry and mode of action that are distinct from the existing drugs. Plant endophytic fungi are a huge source of bioactive natural products with novel structural features and biological activities. Natural products of the mycoepoxydiene family contain an unprecedented structure, 9-oxabicyclo[4.2.1]nona-2,4-diene skeleton. So far, the exact biosynthetic mechanism for this structure has not been reported, although it was thought to be of polyketide origin [9]. The mechanism by which the oxygen bridge is formed within the cyclooctadiene ring is particularly intriguing. The post-polyketide tailoring enzymes involved in the biosynthesis of this rare structural feature could have interesting new features. The isolation and structural elucidation of the new MED analogs provide a new opportunity for the understanding of the biosynthetic mechanism of this family of natural products. Furthermore, these compounds offer the opportunity to investigate the structure–activity relationship. The results revealed that the α,β-unsaturated δ-lactone moiety of the compounds is important for the cytotoxic activity against MDA-MB-435. In addition, compound **4** exhibited activation of AMPK*α* and ACC in NIH/3T3 and 3T3-L1 adipocytes, which indicated that compound **4** might be a potential AMPK agonist. The results suggest that MED analogs could provide lead compounds for structure modifications in development of new agents for AMPK activation. As a key player in the regulation of energy metabolism, AMPK is of central importance in energy metabolism related diseases. AMPK activators hold a great potential in treating metabolic diseases such as type 2 diabetes and obesity.

## 4. Materials and Methods

### 4.1. General Experimental Procedures

The structures were elucidated based on ESI-MS, 1D, and 2D NMR. NMR spectra were recorded on a Bruker Avance III-600 NMR spectrometer (Bruker, Billerica, MA, USA) with TMS as an internal standard. Mass spectrometry analysis was performed using an XTerraMS (Waters, Milford, MA, USA) equipped with an electrospray ionization (ESI) source. HR-ESI-MS data were acquired in *m*/*z* by using a BioTOF^TM^-Q mass spectrometer (Bruker, Billerica, MA, USA) and a Dionex Ultimate 3000 coupled to a Bruker Maxis Q-TOF.

### 4.2. Microbial Strains

Strain A123 was isolated from the foliage of *Kandelia candel* (L.) Druce, a mangrove plant in the Mangrove Nature Conservation Area of Fugong, Fujian Province, China. It was identified as a non-sporulating fungus by traditional morphology. By sequencing the ITS rDNA and comparing it with sequences in GenBank, strain A123 was identified as a *Phomopsis* sp., showing a 98% similarity to *Phomopsis liquidambari* (Accession No. AY 601919) [19]. Strain A818 was screened from a genome shuffling mutagenesis library of strain A123 with enhanced yield of mycoexydiene [16,17].

### 4.3. Culture Conditions and Extraction

All strains derived from *Phomopsis* sp. A123 were maintained on potato dextrose agar (PDA) slants containing 20% (*v*/*v*) stored sea water. For regular cultures, the strains on stock slants were inoculated on PDA plates containing 20% artificial sea water and allowed to grow for 14 days at 28 °C.

Strain A818 was cultured at 28 °C, 180 rpm with 150 L of PDB (Potato Dextrose Broth) medium containing 20% (*v*/*v*) stored sea water. After 14 d cultivation, the mycelia were separated from liquid with ultracentrifugation. The supernatant was collected and extracted with ethyl acetate for three times, ethyl acetate phase was collected together and concentrated under vacuum to afford 60 g of residue.

### 4.4. Structure Isolation and Purification

The crude extract was redissolved in methanol and subjected to medium pressure liquid chromatography (MPLC) over RP-18 silica gel (2 kg, 200–300 mesh, Qingdao Marine Chemical, Inc*.*, Qingdao, China), using a stepwise gradient of 10, 30, 50, 70, and 100% (*v*/*v*) methanol in water. Five fractions (Fr. 1–5) were obtained and further purified by a series of sequential column chromatography on Sephadex LH-20 (40–70 μm, Amersham Pharmacia Biotech AB, Uppsala, Sweden), Lichroprep reversed-phase RP-18 silica gel (40–63 μm, Merck, Darmstadt, Germany), and HPLC (RP-18, Merck). TLC with pre-coated silica gel *GF_254_* plates (0.20–0.25 mm, Qingdao Haiyang Chemical Co. Ltd., Qingdao, China) was used for routing analysis. Compound **1** (1.1 g), compound **2** (2.4 g), compound **3** (0.7 mg), compound **4** (145.7 mg), compound **5** (105.3 mg), compound **6** (107.2 mg), compound **7** (13.8 mg), and compound **8** (10.9 mg).

### 4.5. Cell Culture and Cytotoxicity Assays

NIH/3T3 (mouse embryo fibroblast cell line) was maintained in Dulbecco’s minimal essential medium (DMEM, Gibico, Waltham, MA, USA) supplemented with 10% inactivated fetal bovine serum (FBS, Hyclone, Waltham, MA, USA). 3T3-L1 preadipocytes were cultured in DMEM supplemented with 10% calf serum (Hyclone). Differentiation was induced by treating the cells with differentiation inducers (DMEM) containing 0.5 mM 3-isobutyl-1-methylxanthane (IBMX), 0.25 μM dexa-methasone, 10 μg/mL insulin, and 10% fetal bovine serum for 72 h. More than 90% of the cells expressed the adipocyte phenotype between 8 and 10 days after initiation of differentiation and were used for the experiments. The cells were refed with DMEM supplemented with 10 μg/mL insulin and 10% FBS for the following 48 h and changed every two days. The cell lines were grown in logarithmic growth at 37 °C in a humidified atmosphere consisting of 5% CO_2_ and 95% air.

The cytotoxicity was measured by the MTT (microculture tetrazolium [3-(4,5-dimethylthiazol-2-yl)-2,5]-diphenyl-tetrazalium bromide, Sigma-Aldrich, St. Louis, MO, USA) assay [22,23]. Briefly, the cells plated in the wells of 96-well plates (BD Biosciences, San Jose, CA, USA), were treated in triplicate with various concentrations of compounds for 72 h at 37 °C. After change fresh medium, a 20 μL aliquot of MTT solution (5 mg/mL) was added and incubated for 4 h at 37 °C. 100 μL of triplex solution (10% SDS, 5% isobutanol, 12 mM HCl) was added to each well and incubated overnight at 37 °C. The optical density of each well was measured with a microplate reader (M-3350, Bio-Rad, Hercules, CA, USA) at 595 nm. Growth inhibition rates were calculated with the following equation:

(1) $$\mathrm{Inhibition}\;\mathrm{rate}=\frac{\mathrm{ODcontrol}\;\mathrm{well}-\mathrm{ODtreated}\;\mathrm{well}}{\mathrm{ODcontrol}\;\mathrm{well}}\times 100\%$$

The IC_50_ was defined as the concentration of compound that resulted in a 50% inhibition of growth rate. Data were obtained from five different experiments and present as mean ± SD.

### 4.6. Western Blot Analyses

Cells were lysed in ice-cold RIPA buffer (50 Mm Tris-HCl (pH 7.4); 150 Mm NaCl; 1 mM EDTA; 1% NP-40; 0.25% sodium deoxycholate) with protease inhibitors (Protease inhibitor Cocktail Tablets, Roche, Shanghai, China). Proteins were separated by SDS-PAGE (Bio-Rad) and transferred to Immobilon-P membranes (Millipore, Billerica, MA, USA) and immunoblotted using the following antibodies: anti-AMPK*α* (Cell signaling technology, Danvers, MA, USA, #5831), anti-ACC (Cell signaling technology, #3676), anti-phospho-AMPK*α* (Thr172) (Cell signaling technology, #2535), phospho-ACC (Ser79) (Cell signaling technology, #11818), and anti-*β*actin (Sigma-Aldrich, A5441). The phosphorylation was quantified as the ratio of AMPK*α* and ACC to its total protein level.

## Supplementary Materials

The following are available online, Table S1: ^1^H-NMR (600 MHz) and ^13^C-NMR (150 MHz) data for Compounds **1**–**4** (CD_3_OD), Table S2: ^1^H-NMR (600 MHz) and ^13^C-NMR (150 MHz) data for Compounds **5**–**8** (CD_3_OD), Table S3: Cytotoxicity evaluation of the compounds **1**–**8** (except **3**) against MDA-MB-435 cell clines in vitro.
