# Supplementary material for: Cytotoxic Polyketides with an Oxygen-Bridged Cyclooctadiene Core Skeleton from the Mangrove Endophytic Fungus Phomosis sp. A818
Source: Molecules. 2017 Sep 14;22(9):1547. doi: 10.3390/molecules22091547 (PMC6151419; doi:10.3390/molecules22091547)
Supplement: Supplementary file 1 [file molecules-22-01547-s001.pdf]

Table S1. ^{1}H-NMR (600 MHz) and ^{13}C-NMR (150 MHz) data for Compounds **1-4** (CD_3OD)[a]

	Compound **1**		Compound **2**		Compound **3**		Compound **4**	
No.	δ_H (mult.,*J* in Hz)[b]	δ_C	d_H (mult.,*J* in Hz)	d_C	d_H (mult.,*J* in Hz)	d_C	d_H (mult.,*J* in Hz)	δ_C
1	/	162.1s	/	163.1s	/	163.2s	/	169.4s
2	6.24(d, 9.7)	125.1d	6.15(d,9.7)	123.1d	6.24(d, 9.7)	123.8d	2.61(t,)	25.2t
3	7.05(dd, 6.0,9.7)	140.2d	7.06(dd,6.1,9.6)	144.1d	7.14(dd,6.1, 9.7	141.1d	2.20(m)	25.1t
4	5.09(dd, 2.3,5.9)	63.2d	4.09(dd,1.7,5.9)	62.1d	5.24(dd,2.2, 6.1)	62.5d	5.05(m)	66.7d
5	4.51(dd,2.3,11.0)	77.6d	4.35(dd,2.3,10.8)	79.4d	4.78(dd,2.2, 11.2)	77.8d	4.39(d,11.0)	79.5d
6	3.08(m)	50.0d	3.08(ddd,2.0,7.0,10.8)	51.6d	3.17(m)	49.2d	2.98(m)	52.6d
7	4.30(t,6,6)	75.9d	4.45(dd,5.6,11.3)	76.9d	4.30(t,5.8)	71.2d	4.28(t,6.1,5.9)	75.9d
8	6.07(bdd,5.9, 10.7)	136.9d	6.14(dd,4.8,7.6)	137.2d	6.05(dd,5.2, 10.8)	137.4d	6.06(m)	137.3d
9	5.90(m)	126.3d	5.98(m)	126.2d	5.87(dd,6.9, 10.20	124.8d	5.88(m）	126.1d
10	5.94(m)	124.4d	5.92(m)	124.8d	5.95(dd,7.0, 11.9)	123.9d	5.92(m)	124.4d
11	6.12(dd,4.2,7.4)	137.5d	6.28(dd,5.7,10.3)	137.3d	6.08(d,11.5)	139.2d	6.09(m)	137.5d
12	4.33(d,4.5)	86.4d	4.33(dd,2.0,5.0)	86.1d	/	106.0s	4.31(d,4.5)	86.4d
13	3.01(m)	52.6d	3.00(ddd,1.3,7.1,8.3)	51.8d	2.72((quint, 7.1)	51.3d	2.91(m)	51.2d
14	1.15(d,6.8)	14.1q	1.15(d,7.1)	14.3q	1.11(d,7.2)	8.2q	1.13(d,6.9)	14.1q
	/	170.0s			/	170.0s	/	170.1s
Ac-4	2.04(s)	20.7q			2.04(s)	19.2q	2.15(s)	21.0q

[a] ^{1}H-NMR and ^{13}C-NMR spectra were obtained at 500 MHz and 125 MHz, respectively, and were recorded in CD_3OD at room temperature.

[b] Unless otherwise indicated, all proton signals integrate to ^{1}H.

Table S2. ^{1}H-NMR (600 MHz) and ^{13}C-NMR (150 MHz) data for Compounds **5-8** (CD_3OD)[a]

	Compound **5**		Compound **6**		Compound **7**		Compound **8**	
No.	δ_H (mult.,*J* in Hz)[b]	δ_C	δ_H (mult.,*J* in Hz)	δ_C	δ_H (mult.,*J* in Hz)	δ_C	δ_H (mult.,*J* in Hz)	δ_C
1	/	170.2s	/	170.4s	/	176.3s	/	179.2s
2	6.28(d, 5.4)	119.0d	6.33d	120.1d	2.48(m,2H)	28.1t	2.62(m,2H)	28.0t
3	7.68(d, 5.5)	144.3d	7.97(d,6.1)	140.1d	2.28(m),2.06(m)	23.9t	2.49(m),2.22(m)	23.6t
4	/	150.2s	/	150.9s	4.56(ddd, 2.3,8,11.8)	80.1d	4.59(dd,5.9, 13.6)	82.8d
5	5.57(d, 10.6)	114.7d	5.90(d, 11.8)	114.5d	5.13(dd, 2.5,10.5)	72.6d	3.61(d,11.1)	71.1d
6	3.78(ddd, 3.5,7,11)	53.8d	3.64(ddd, 2.8,7.4,11.2)	54.3d	2.96(ddd, 6,7,10.5)	51.5d	2.77(m)	53.2d
7	4.48(dd,3.5,8.1)	83.0d	4.46(dd, 2.9,5.2)	83.3d	4.41(t, 6,6)	78.1d	4.41(dd,6.1, 12.1)	77.2d
8	6.19(dd,6.5,11.5)	136.4d	6.18(dd,5.2,10.2)	136.0d	6.21(dd, 5.3,11.4)	137.6d	6.31(dd,5.7, 9.4)	138.0d
9	5.92(m)	125.3d	5.97(m)	125.5d	5.91(m)	126.2d	5.87(m)	125.5d
10	5.95(m)	124.8d	5.95(m)	124.9d	5.87(m)	125.0d	5.93(m)	124.2d
11	6.25(dd, 5.0,11.6)	137.3d	6.27(dd,1.7,10.0)	137.5d	6.04(dd, 4.6, 11.8)	136.9d	6.10(dd,4.2, 8.2)	136.7d
12	4.32(dd, 4.1,8.5)	84.8d	4.33(dd,3.9,5.1)	84.7d	4.21(d, 4.5)	86.2d	4.26(d,4.4)	86.6d
13	2.88(ddq, 3.4,7.3,10.7)	51.7d	2.85(ddq,3.8,7.3,11.1)	51.5d	2.71(ddq, 4.5,6.9,13.9)	52.3d	2.85(m)	53.2d
14	1.06(d,7.2)	14.2q	1.05(d,7.3)	14.2q	0.95(d, 7.0)	14.4q	1.15(d,7.0)	12.9q
					/	170.5s		53.2d
Ac-4					2.08(s)	23.9q		12.9q

[a] ^{1}H-NMR and ^{13}C-NMR spectra were obtained at 500 MHz and 125 MHz, respectively, and were recorded in CD_3OD at room temperature.

[b] Unless otherwise indicated, all proton signals integrate to ^{1}H.

Table S3. Cytotoxicity evaluation of the compounds **1-8** (except **3**) against MDA-MB-435 cell clines *in vitro*

Compound	IC_{50} (μM)
1	7.85±0.60
2	14.61±1.15
4	>500 (12.24%)
5	>50 (6.35%)
6	>50 (7.53%)
7	>500 (33.94%)
8	>50 (8.51%)

Data are presented as mean ± SD (n = 5). Compounds **1-8** dissolved in DMSO were diluted with culture medium containing 0.1% DMSO, respectively. The control cells were treated with culture medium containing 0.1% DMSO. Cisplatin was used as a positive control. When 50% inhibition could not reached at the highest concentration, and then inhibition of growth rate is given in parentheses.
